# Supplementary material for: Peptide-Conjugated Phosphorodiamidate Morpholino Oligomers for In Situ Live-Cell Molecular Imaging of Dengue Virus Replication
Source: Int J Mol Sci. 2020 Dec 4;21(23):9260. doi: 10.3390/ijms21239260 (PMC7730579; doi:10.3390/ijms21239260)
Supplement: Supplementary file 1 [file ijms-21-09260-s001.pdf]

x

(A)

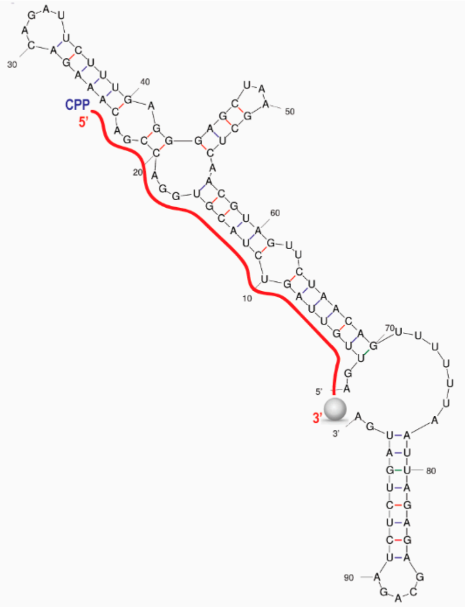

(B)

```
                  10        20        30        40        50        60        70
               5'....|....|....|....|....|....|....|....|....|....|....|....|....| 3'
DENV-2 S221    AGUUGUUAGUCUACGUGGACCGACAAAGACAGAUUCUUUGAGGGAGCUAAGCUCAACGUAGUUCUAACAG
5'SL PPMO      TCAACAATCAGATGCACCTGGCTG----------------------------------------------
ZIKV H/PF/2013 AGUUGUUGAUCUGUGUGAAUCAGACUGCGACAGUUCGAGUUUGAAGCGAAAGCUAGCAACAGUAUCAACA
```

(C)

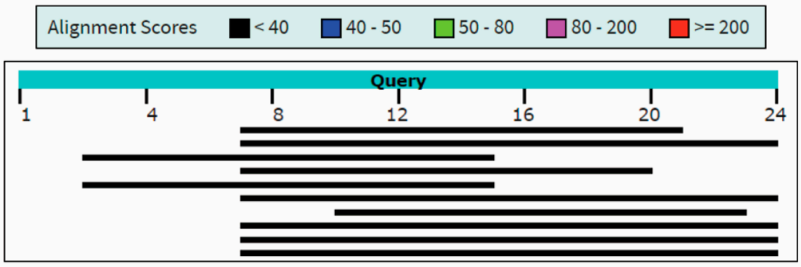

	Description	Max Score	Total Score	Query Cover	E value	Per. Ident	Accession
☑	Homo sapiens zinc finger protein 653 (ZNF653), mRNA	28.2	28.2	58%	22	100.00%	NM_138783.4
☑	Homo sapiens slingshot protein phosphatase 1 (SSH1), transcript variant 2, mRNA	26.3	26.3	70%	87	94.12%	NM_001161330.2
☑	Homo sapiens G-patch domain containing 2 like (GPATCH2L), transcript variant 1, mRNA	26.3	26.3	54%	87	100.00%	NM_017926.4
☑	Homo sapiens zinc finger protein 264 (ZNF264), mRNA	26.3	26.3	54%	87	100.00%	NM_003417.5
☑	Homo sapiens zinc finger protein 512 (ZNF512), transcript variant 1, mRNA	26.3	26.3	54%	87	100.00%	NM_032434.4
☑	Homo sapiens slingshot protein phosphatase 1 (SSH1), transcript variant 1, mRNA	26.3	26.3	70%	87	94.12%	NM_018984.4
☑	Homo sapiens colony stimulating factor 2 receptor subunit beta (CSF2RB), mRNA	26.3	26.3	54%	87	100.00%	NM_000395.3
☑	PREDICTED: Homo sapiens slingshot protein phosphatase 1 (SSH1), transcript variant X7, r	26.3	26.3	70%	87	94.12%	XM_011538501.3
☑	PREDICTED: Homo sapiens slingshot protein phosphatase 1 (SSH1), transcript variant X5, r	26.3	26.3	70%	87	94.12%	XM_011538500.3
☑	PREDICTED: Homo sapiens slingshot protein phosphatase 1 (SSH1), transcript variant X3, r	26.3	26.3	70%	87	94.12%	XM_017019491.2

Figure S1. Specificity of 5′SL peptide-conjugated phosphorodiamidate morpholino oligomer (PPMO) to Dengue virus RNA. (**A**) Structure of the stem-loop region at the 5′terminus (5′SL) genome region of Dengue virus serotype-2 (DENV2) mouse-adapted S221 strain (GenBank Accession No. HQ541799.1). The structure was drawn using Mfold Web Server [1]. The red line represents the 5′SL PPMO in its correct orientation of binding to the 5′SL structure. The ball represents the fluorophore (6-carboxyfluorescein) tag. (**B**) Sequence alignment of 5′SL PMO with the target 5′SL region of DENV2 and Zika virus (ZIKV) French Polynesian H/PF/2013 (GenBank Accession No. KJ776791.2) strain. The sequences mismatched to the 5′SL PMO are underlined in red. (**C**) Potential mRNA binding sites of 5′SL PPMO within the cell. Top ten results of BLASTN search for potential binding sites of 5′SL PMO against Genbank human mRNA database. Of the top hits, none exhibited complementarity to the full 24-mer 5′SL PMO sequence. The top hits exhibited only maximum alignment scores < 40.

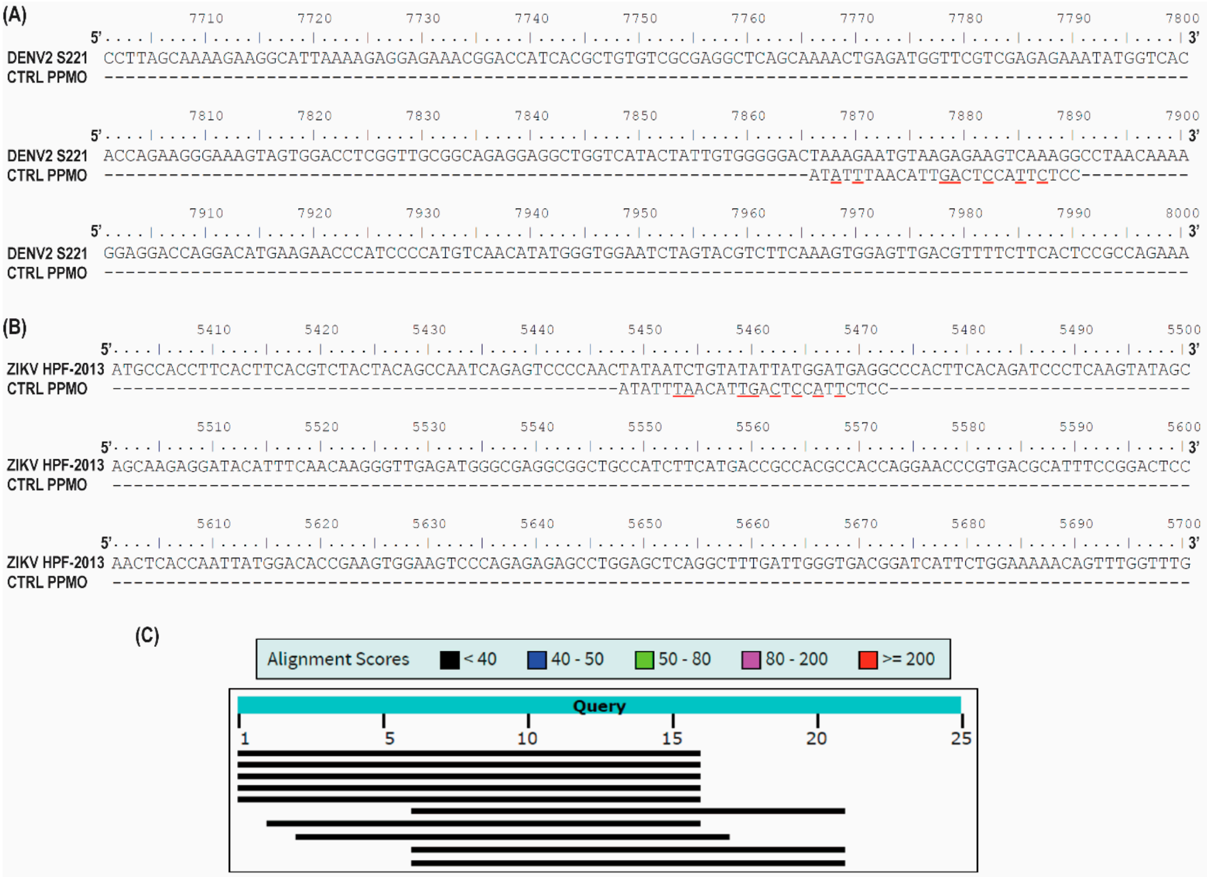

	Description	Max Score	Total Score	Query Cover	E value	Per. Ident	Accession
☑	PREDICTED: Homo sapiens shroom family member 1 (SHROOM1), transcript variant X4, ml	32.2	32.2	64%	1.6	100.00%	XM_024454367.1
☑	PREDICTED: Homo sapiens SET domain containing 5 (SETD5), transcript variant X5, mRNA	32.2	32.2	64%	1.6	100.00%	XM_017006768.2
☑	PREDICTED: Homo sapiens shroom family member 1 (SHROOM1), transcript variant X6, mi	32.2	32.2	64%	1.6	100.00%	XR_427704.3
☑	PREDICTED: Homo sapiens shroom family member 1 (SHROOM1), transcript variant X5, ml	32.2	32.2	64%	1.6	100.00%	XM_006714534.3
☑	PREDICTED: Homo sapiens shroom family member 1 (SHROOM1), transcript variant X1, ml	32.2	32.2	64%	1.6	100.00%	XM_005271885.4
☑	Homo sapiens ciliogenesis and planar polarity effector 1 (CPLANE1), transcript variant 1, mR	30.2	30.2	60%	6.3	100.00%	NM_001384732.1
☑	Homo sapiens RAB14, member RAS oncogene family (RAB14), mRNA	30.2	30.2	60%	6.3	100.00%	NM_016322.4
☑	Homo sapiens gamma-aminobutyric acid type A receptor subunit pi (GABRP), transcript varia	30.2	30.2	60%	6.3	100.00%	NM_014211.3
☑	PREDICTED: Homo sapiens ciliogenesis and planar polarity effector 1 (CPLANE1), transcrip	30.2	30.2	60%	6.3	100.00%	XR_002956171.1
☑	PREDICTED: Homo sapiens ciliogenesis and planar polarity effector 1 (CPLANE1), transcrip	30.2	30.2	60%	6.3	100.00%	XM_024446186.1

Figure S2. Potential binding sites of control (CTRL) peptide-conjugated phosphorodiamidate morpholino oligomer (PPMO) within the cell. **(A**,**B)** Sequence alignment of non DENV-targeted unrelated control (CTRL) PPMO with (**A**) Dengue Virus serotype-2 (DENV-2) mouse-adapted S221 strain (GenBank Accession No. HQ541799.1) and (**B**) Zika virus (ZIKV) French Polynesian H/PF/2013 strain (GenBank Accession No. KJ776791.2). Regions with mismatched complementarity are underlined in red. (**C**) Potential mRNA binding sites of CTRL PPMO within the cell. Top ten results of BLASTN search for potential binding sites of CTRL PMO against Genbank human mRNA database. Of the top hits, none exhibited complementarity to the full 25-mer CTRL PMO sequence. The top hits exhibited only maximum alignment scores < 40.

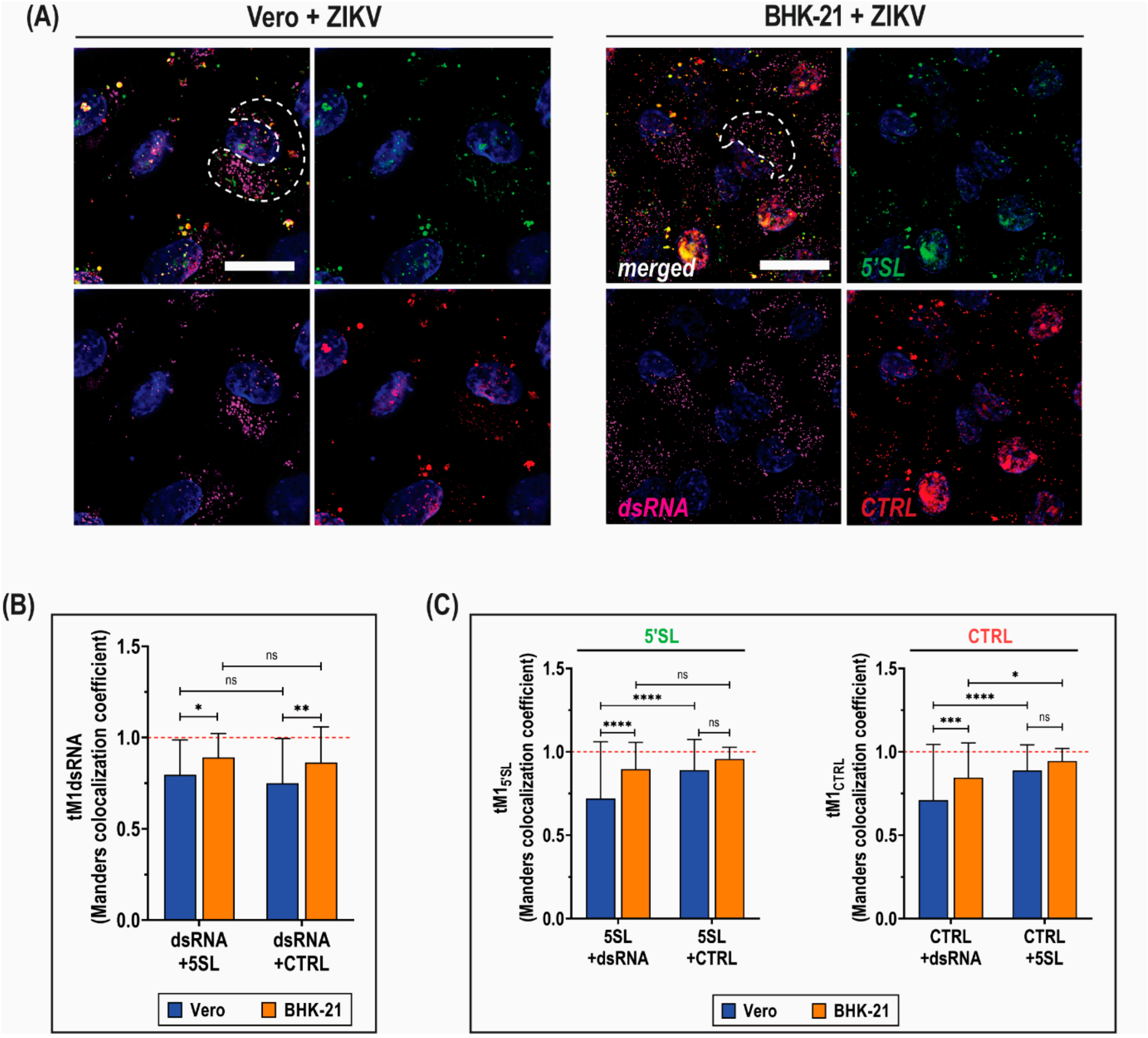

Figure S3. Colocalization of PPMOs with replicating ZIKV genome. (**A**) Representative deconvoluted fluorescence images of monkey kidney (Vero) and baby hamster kidney (BHK-21) cells incubated with both targeted 5SL and non-targeted CTRL PPMOs (10 μM each) at 48 h post-infection with 1 MOI Zika virus (ZIKV) H/PF/2013 strain. The cells were subsequently incubated with J2 monoclonal antibody against double-stranded RNA (dsRNA), which was used to tag virus replication complexes within cells. Examples of regions of interest (ROIs) used for signal colocalization analyses are outlined in white dashed lines. (**B**,**C**) Thresholded Manders coefficients (tM1), which represents the degree of colocalization of two fluorescence signals, calculated from fluorescence images in (A). (**B**) tM1 values from colocalization of viral replication vesicle packets (VPs)with either 5′SL or CTRL PPMO in ZIKV-infected cells evaluated at.48 h after infection (**C**) tM1 values from colocalization of either 5′SL or CTRL PPMO with viral replication VPs at 48 h after infection. Columns represent mean values, and error bars represent standard deviation. The red dashed line indicates ratio = 1. Mean values were compared with two-way ANOVA. **, p <0.005; ***, p < 0.0005; ****, p < 0.0001; *ns*, not significant. Scale bar = 20 μm.

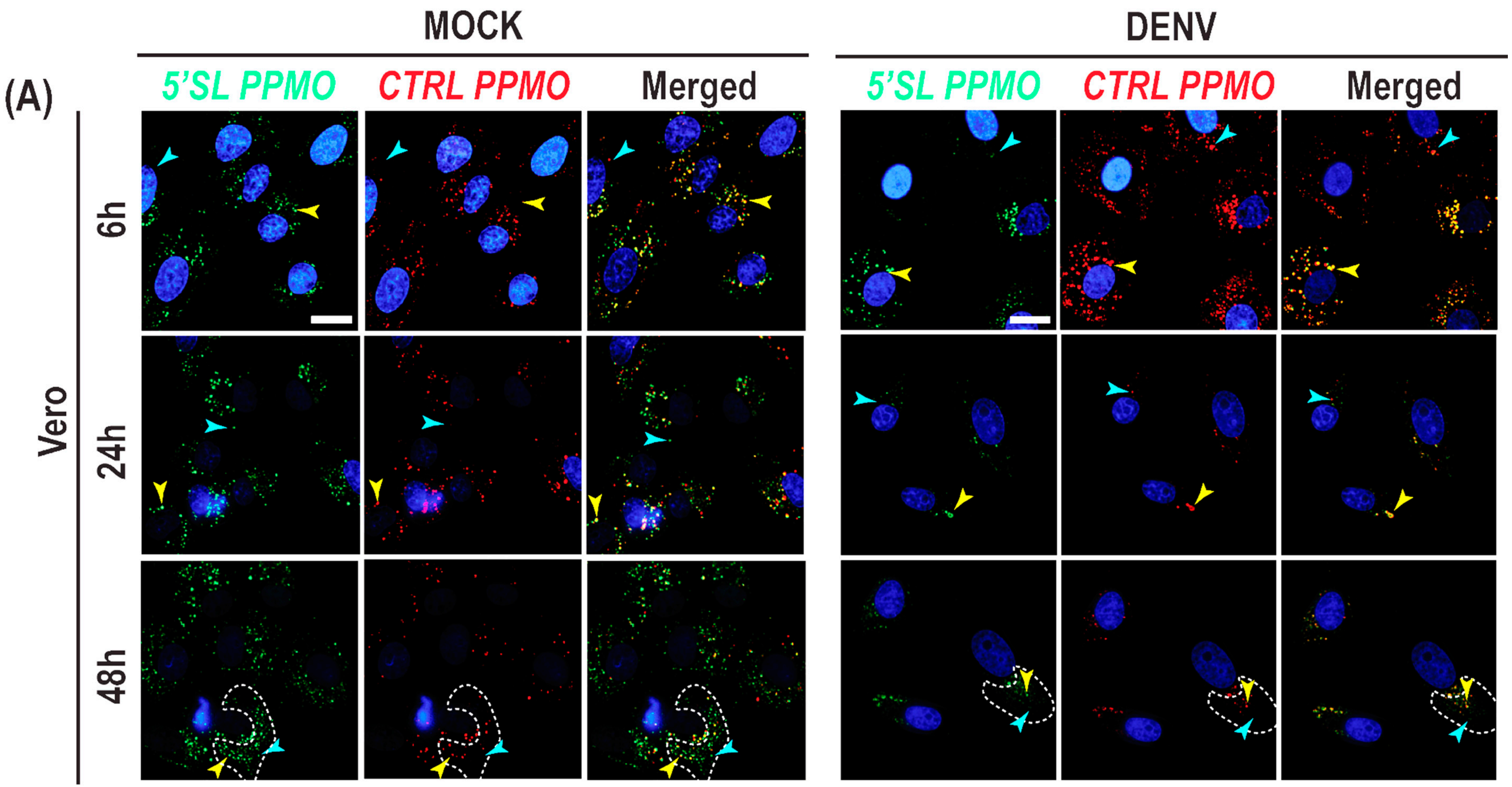
MOCK
DENV
(A)
5'SL PPMO
CTRL PPMO
Merged
5'SL PPMO
CTRL PPMO
Merged
Vero
6h
24h
48h

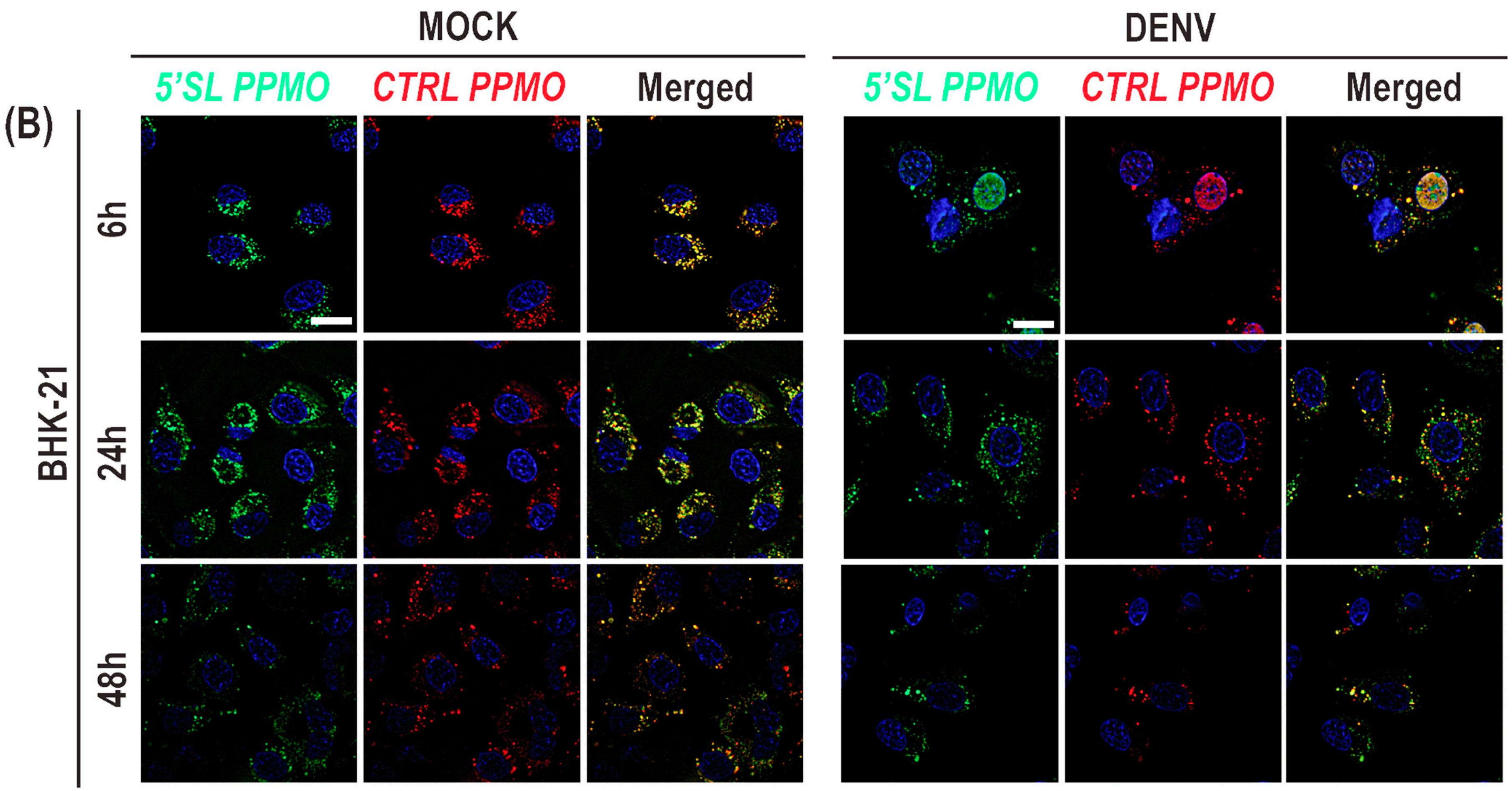
MOCK
DENV
5'SL PPMO
CTRL PPMO
Merged
5'SL PPMO
CTRL PPMO
Merged
(B)
BHK-21
6h
24h
48h

Figure S4. Representative fluorescent live-cell images following incubation with 5′SL and CTRL PPMOs. Deconvoluted fluorescence images of (**A**) Vero and (**B**) BHK-21 cells that were either infected with Dengue virus serotype-2 (DENV2) mouse-adapted S221 strain or mock-infected, incubated with 5′SL PPMO and CTRL PPMO (10 μM each) for 10 min and subsequently imaged at 6, 24, and 48 h after PPMO incubation. PPMOs were added at 1 h post-infection. FAM-labeled 5′SL PPMO and Lissamine Red-labeled CTRL PPMO are imaged in green and red fluorescence channels, respectively, with probes concentrating in intracellular compartment and visualised as punctae. Representative punctae areas where the PPMOs colocalize are indicated by the yellow arrows. Areas where the PPMOs do not colocalize are indicated by the blue arrows. Scale bar = 20 μm.

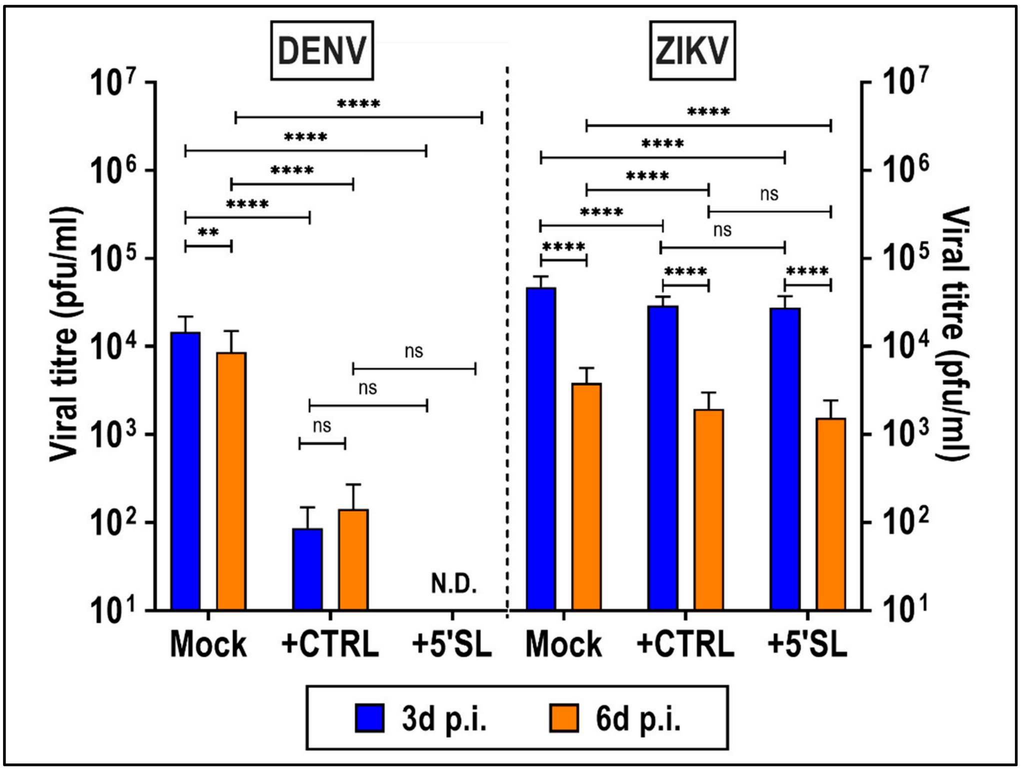

Figure S5. Antiviral effect of 5′SL PPMO against Dengue virus replication. Comparison of viral titres in culture supernatants harvested from Vero cells infected with either Dengue virus (DENV) mouse-adapted S221 strain or Zika virus (ZIKV) French Polynesian H/PF/2013 strain. Infected cells were treated with 10 μM of either 5′SL or SCR PPMO prior to virus infection. Viral titres in supernatants harvested at 3 or 6 days after PPMO addition were determined by plaque-formation assay (PFA) using Vero cells and reported as plaque-forming units (pfu) per unit volume. Columns represent mean values, while error bars represent SD. Mean values were compared by two-way ANOVA. **, p <0.005; ****, $p < 0.0001$; *ns*, not significant. *N.D.*, none detected.

References

1. Zuker, M. Mfold web server for nucleic acid folding and hybridization prediction. *Nucleic. Acids. Res.* **2003,** 31, 3406–3415.
